# Finsen Light in the Treatment of Lupus Vulgaris

**Published:** 1908-07-25

**Authors:** 


### DERMATOLOGY.

### FINSEN LIGHT IN THE TREATMENT OF LUPUS VULGARIS.

The Finsen method of treatment consists in the application of light to diseased areas of the skin. It is mainly used in the treatment of lupus vulgaris. It has long been known that light has a bactericidal action, or, at any rate, that it inhibits the growth of micro-organisms; and it was this fact which first suggested its use as a means of treatment for lupus vulgaris. It is now known, however, that it is not this bactericidal property of light which renders its local application efficacious in the cure of lupus, but that the curative effect is the result of the inflammatory reaction which it produces in the tissues exposed to its influence. This property which light possesses of producing inflammatory reaction is also a matter of common knowledge, and it is well seen in sunburn.

In order to obtain a sufficient action it is found necessary to concentrate the light by means of lenses upon the parts to be treated. Light, however, from whatever source, whether directly from the sun or artificial light, is accompanied by heat. But the action of the heat rays is too destructive and too painful for therapeutic purposes, so that, while concentrating the light, means must be devised for cutting off the heat rays. Originally, Finsen made use of the direct rays of the sun. These were focussed on to the part by means of a series of telescopic lenses, and the apparatus was filled with blue-tinted water, in order to cut off the heat rays. But it has been found more convenient to use artificial light, and one now employs a very powerful electric carbon-arc as a source of light. The rays from the arc lamp are focussed on to the part by means of a series of quartz lenses (quartz having the property of cutting off the heat rays to some extent), and a water-cooling system is employed. As a further precaution it is necessary to remove the barrier to the penetration of the light rays which is offered by the red colouring matter of the blood in the tissues under treatment, This is effected by the employment of a water-cooled quartz compressor, which is kept constantly pressed on the part while the exposure is taking place.

The lamp now in most general use is that known as the Finsen-Reyn. The patient lies on a couch, and the rays are focussed on to the area to be treated. The nurse, during the whole exposure, keeps the spot that is being exposed free from blood by constant pressure with the compressor.

It is found that the best results are obtained when the inflammatory reaction is greatest, and, generally, an exposure of from half an hour to one hour is given. During the application the patient feels no pain beyond that produced by the pressure of the compressor. A few hours later the part that has been exposed becomes red, hot, and smarting. This inflammation lasts for about a week and then subsides. Blistering and crusting may take place. When the inflammation has subsided a second exposure may be given to the same spot, and the same area may require several such applications before the lesion disappears. When it does there is left only a very fine and supple scar. Only a small area—at most a space half an inch in diameter—can be exposed at one time, for the part must be almost within the focus of the rays. For a large area of lupus, therefore, very many sittings are necessary. This long duration of the treatment, the expensiveness of the apparatus, the need of constant supervision by a nurse, and the tedium of the repeated long sittings to the patient must be regarded as decided drawbacks to the method. On the other hand, and particularly for lupus on the face, no other method (except excision, which is suitable for limited areas away from the face) can compare with the Finsen light treatment in efficiency and in cosmetic results.

The most suitable cases for light treatment are early non-ulcerated patches upon the face. If the patch is of very large extent it is very difficult for the Finsen treatment to keep pace with the extension of the disease, slow as it often is. Where the lesions are warty or crusted Finsen light cannot be properly applied, and a preliminary treatment by $x$-rays—in carefully measured doses, giving a Sabouraud pastille dose once in three or four weeks—is the best method of getting rid of the crusts or of the warty growth, and of thus reducing the lupus to its primitive elements, the apple-jelly nodules. Then the Finsen light may be applied as in a purely nodular lesion. Ulcerating lesions and mucous membrane lesion are also better treated by $x$-rays than by Finsen light.

